# The effects of trace element supplementation on glycolipid metabolism in PCOS: a systematic review and meta-analysis

**DOI:** 10.3389/fnut.2025.1683556

**Published:** 2025-10-09

**Authors:** Liuzhen Yang, Yi Gao, Wei Zhao, Yuwen Qi, Xinru Duo, Huixia Wang

**Affiliations:** ^1^The Third Affiliated Hospital of Henan University of Chinese Medicine, Zhengzhou, China; ^2^The Second Clinical Medical College of Henan University of Chinese Medicine, Zhengzhou, China

**Keywords:** meta-analysis, glucose and lipid metabolism, trace elements, polycystic ovarian syndrome, sex hormones

## Abstract

**Objectives:**

A common endocrine and metabolic condition affecting women of reproductive age is polycystic ovarian syndrome (PCOS). The link between trace elements and PCOS has drawn more attention in recent years. However, the complete therapeutic potential of trace element supplementation in PCOS therapy is still unknown. Thus, the purpose of this study is to look at how supplementing with four trace elements– calcium, chromium, selenium and magnesium–may affect the metabolism of glycolipids and other clinical outcomes in women with PCOS.

**Methods:**

To find randomized controlled trials (RCTs), a comprehensive literature search was carried out up until May 2025 using four internet databases: the Cochrane Library, Web of Science, Embase, and PubMed. Intervention studies that evaluated the impact of calcium, chromium, selenium and magnesium supplementation on important outcomes such as blood glucose levels, lipid profiles, oxidative stress markers, inflammatory responses, sex hormone concentrations, and body weight in PCOS patients met the inclusion criteria. Heterogeneity between studies was evaluated using the I^2^ statistic, a result of more than 50% indicates significant heterogeneity.

**Results:**

A total of 25 RCTs with a combined sample size of 1,600 PCOS patients were considered. The results showed a significant decrease in fasting blood glucose levels (SMD = −0.79, 95% CI: −1.11 to −0.46). Both insulin resistance as determined by homeostasis model assessment of β-cell function (SMD = −0.68, 95% CI: −1.00 to −0.36) and fasting insulin levels were significantly lower (SMD = −0.58, 95% CI: −0.90 to −0.26). Additionally, it was discovered that taking supplements of selenium increased the QUICKI index (SMD = 0.53, 95% CI: 0.15 to 0.91) and considerably decreased fasting insulin concentrations (SMD = −0.32, 95% CI: −0.63 to −0.01). Magnesium supplementation did not show statistically significant impacts on any glucose metabolic measures, however neither fasting plasma glucose nor HOMA-IR showed any statistically significant effects. Chromium supplementation was observed to significantly lower levels of very-low-density lipoprotein (SMD = −0.59, 95% CI: −0.91 to −0.27) and triglycerides (SMD = −0.59, 95% CI: −0.91 to −0.27) in relation to lipid metabolism. Other lipid measures, such as total cholesterol, low-density lipoprotein cholesterol, and high-density lipoprotein cholesterol, did not, however, show any statistically significant changes. Supplementing with magnesium or selenium had no statistically significant effects on any of the lipid metabolic markers. Calcium supplementation was observed to significantly lower levels of nitric oxide (SMD = −0.45, 95% CI: −0.84 to −0.06) and malondialdehyde (SMD = −0.76, 95% CI: −1.15 to −0.36) in relation to oxidative stress markers. Malondial-dehyde levels (SMD = −1.69, 95% CI: −3.10 to −0.28) and high-sensitivity C-reactive protein levels (SMD = −0.65, 95% CI: −1.05 to −0.24) were shown to be considerably reduced by chromium supplementation. Furthermore, a noteworthy rise in total antioxidant capacity was linked to it (SMD = 1.47, 95% CI: 1.02 to 1.92). Malondial-dehyde and inflammatory cytokines did not show any statistically significant changes, while selenium supplementation was shown to significantly increase total antioxidant capacity (SMD = 0.55, 95% CI: 0.16 to 0.95). None of the oxidative stress markers were significantly regulated by magnesium; The levels of sex hormones, including follicle-stimulating hormone, luteinizing hormone, dehydroepiandrosterone, sex hormone-binding globulin, testosterone, total testosterone, and the free androgen index, did not significantly improve with supplementation of chromium, calcium, magnesium, and selenium; Across all trace element supplementation regimens, no statistically significant variations were seen in weight-related measures, including body weight, waist circumference, hip circumference, and body mass index. Further high-quality randomized controlled studies are necessary to validate the low efficacy of calcium and magnesium, across the majority of outcome measures.

**Conclusion:**

In PCOS patients, chromium has a clear therapeutic benefit in reducing oxidative stress, dyslipidemia, and glucose metabolic disorders. Selenium has demonstrated promise in raising antioxidant capacity and boosting insulin sensitivity.

## 1 Introduction

One of the most prevalent endocrine disorders affecting women of reproductive age is polycystic ovarian syndrome (PCOS), which is believed to affect 10%–13% of women. It seriously impairs women’s health and socioeconomic progress (1). Among the many clinical manifestations of PCOS include infertility, irregular menstruation, acne, and hirsutism (2). Research indicates that insulin resistance and dysregulated lipid metabolism are two significant pathophysiological processes that support the onset and progression of PCOS (3). Insulin resistance-induced hyperinsulinemia intensifies the stimulatory effect of luteinizing hormone (LH) on ovarian stromal cells, hence enhancing androgen synthesis. These pathophysiological changes result in unique clinical manifestations, including follicular development anomalies, ovulatory failure, and hyperandrogenism (4). Meanwhile, it has been shown that elevated insulin levels prevent the liver from producing sex hormone-binding globulin (SHBG), which impacts steroid hormone transport and bioavailability (5). The concentration of free testosterone rises, aggravating endocrine malfunction and perpetuating hormonal disorders (6). Conversely, rising triglyceride levels, decreasing HDL cholesterol, and elevated concentrations of tiny dense LDL particles are the hallmarks of dyslipidemia (7). In addition to mutually enhancing insulin resistance, it also contributes to the onset of endothelial dysfunction and chronic low-grade inflammation (8),greatly increases the risk of type 2 diabetes in persons with PCOS (9), nonalcoholic fatty liver disease (10), and cardiovascular diseases (11). The combined effects of many metabolic abnormalities are a major cause of the phenotypic heterogeneity and the long-term progression of PCOS.

The potential therapeutic application of trace elements in the management of PCOS is attracting increasing attention from scientists. According to recent research, the pathophysiology of PCOS is inherently linked to insulin resistance, oxidative stress, endocrine dysregulation, and impaired reproductive function (12). Trace elements have a variety of regulatory effects by focusing on these pathological processes. Calcium supplementation reduces the risk of long-term cardiovascular issues by improving significant clinical indications such hirsutism, hyperandrogenism, and irregular menstruation via changing intracellular calcium signaling pathways (13). Insulin resistance is one of the primary pathogenic processes in PCOS. Chromium supplementation increases insulin sensitivity and significantly lowers insulin resistance, which in turn lowers free testosterone, fasting insulin, and body mass index (BMI) (14). Oxidative stress is one of the primary pathogenic causes in the pathological state of PCOS. By modulating the neuroendocrine system and lowering oxidative stress responses, magnesium helps PCOS patients’ mental health and general quality of life (15). In infertile PCOS patients undergoing IVF therapy, selenium, an essential antioxidant, enhances glucose metabolic homeostasis and reduces malondialdehyde (MDA) levels, a confirmed indicator of oxidative stress. This, in turn, boosts ovarian and systemic reproductive antioxidant capacity (16). The aforementioned studies have shed some light on the connections between trace elements and the pathogenic mechanisms of PCOS. However, additional validation through large, multicenter clinical studies is required to develop a comprehensive trace element-based intervention plan for clinical practice.

Regarding the impact of trace element supplementation on PCOS results, there is currently conflicting data (17). This study does a comprehensive review and meta-analysis of the body of current literature to ascertain the precise effects of calcium, chromium, magnesium and selenium on clinical symptoms and biochemical markers in PCOS patients. This offers a scientific foundation for enhancing comprehensive PCOS treatment plans.

### 1.1 Study design

This systematic review and meta-analysis followed the PRISMA (Preferred Reporting Items for Systematic Reviews and Meta-Analyses) guidelines (18). The PROSPERO registration number for this study was CRD420251001972.

### 1.2 Search strategy

Two researchers conducted a systematic search from inception to May 1, 2025, using text keywords and medical subject headings (MeSH). We conducted a comprehensive search of each of the four online databases: PubMed, Embase, Web of Science, and the Cochrane Library. The following terms were part of the search strategy: (1) words connected to PCOS, like “PCOS” and “Polycystic ovarian syndrome”; (2) terms linked to trace elements, like “trace element,” “calcium,” “chromium,” “magnesium,” “selenium”; (3) terms associated with RCTs, like “Randomized controlled trial,” “Placebo,” and “RCT.” To make sure all relevant publications were found, a thorough search of references listed in published me-ta-analyses was also carried out. Supplementary Table 1 contains the comprehensive search details.

### 1.3 Study selection

The following PICO (Population, Intervention, Comparison, and Outcome) elements were set as inclusion criteria: (1) Population: women with PCOS; (2) Intervention: women with PCOS receiving trace element supplementation; (3) Control group: PCOS women with oral placebo; (4) Outcome: all PCOS-related parameters. Studies were excluded under the following circumstances. (1) Reviews, case reports, conference abstracts, as well as animal and cellular experiments. (2) Full-text articles were unavailable, or data could not be extracted. (3) The intervention group was composed of trace elements in combination with other nutritional supplements or pharmaceutical agents. Furthermore, in cases where the same study was reported across multiple articles, the article containing the most comprehensive data set was selected.

### 1.4 Data extraction and quality assessment

To collect data and evaluate the quality of the final included studies, two researchers, Liuzhen Yang and Yi Gao, worked independently. In cases of disagreement, a third researcher, Huixia Wang, was consulted to reach consensus. The first author’s name, the year the study was published, the study’s location, the intervention and control group sample sizes, the age of PCOS women, the trace element supplementation dosage, the length of follow-up, and the effect sizes of outcome measures at baseline and after the intervention were all extracted.

The methodological quality of the included studies was evaluated using the Cochrane Risk of Bias Tool. If a study satisfied four or more of the seven preset criteria, it was classified as low risk of bias; if it satisfied fewer than four, it was classified as high risk. Supplementary Table 2 displays the exact quality evaluation findings for each research.

### 1.5 Statistical analyses

If data on the changes were not readily available, we extracted the pertinent data at baseline and after the intervention and used the following formula to calculate the mean and corresponding standard deviation (SD) of the changes in the data before and after treatment from the literature (19):


$$\begin{array}{l} mean\;=\;mean_{end}\;-\;mean_{baseline}\\ \Delta SD\;=\;\sqrt{SD_{end}^{2}\text{ + }SD_{baseline}^{2}\;-\;2\;\times \;R\;\times \;SD_{end}\;\times \;SD_{baseline}} \end{array}$$

Study heterogeneity was assessed using the I^2^ statistic and the Cochrane Q test. Heterogeneity was considered statistically significant if the I^2^ value was more than 50% or the *p*-value was less than 0.1. A fixed-effects model was used when heterogeneity was not substantial, while a random-effects model was used when heterogeneity was considerable. The data are presented using standardized mean differences (SMD) with 95% confidence intervals (CI). Numerous confounding factors, such as BMI, trace element dose, and duration of intervention, may affect the study’s findings. Publication bias was assessed using Egger’s regression test. All statistical analyses were performed using RevMan 5.4 (Cochrane Collaboration, London, United Kingdom, 2020).

## 2 Materials and methods

### 2.1 Study selection

**FIGURE 1:**
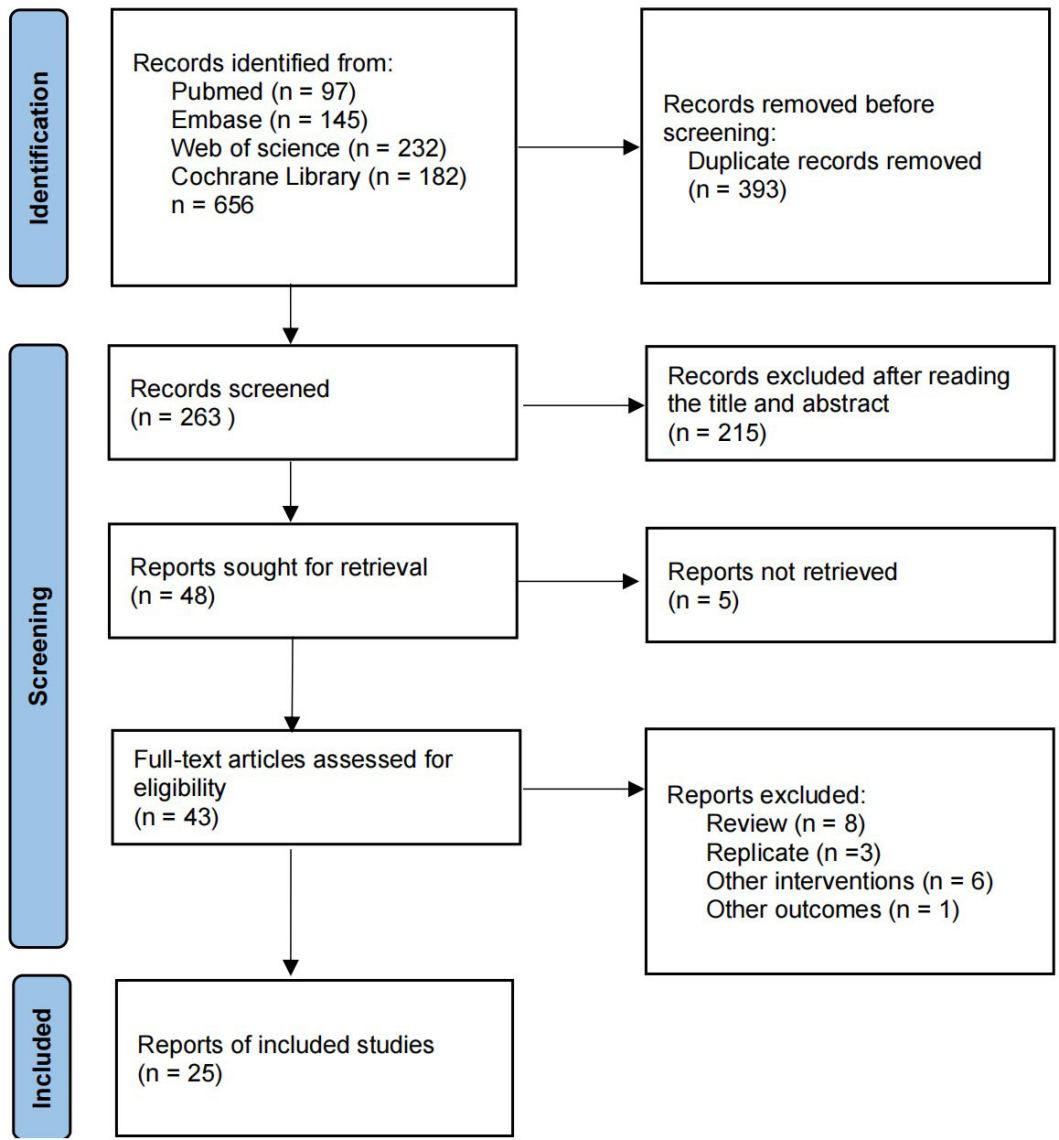
PRISMA flow diagram of the study selection process.

We searched four online databases for 656 relevant articles, including 393 duplicates. Of the remaining 263 articles, 215 were excluded after reading the titles and abstracts, leaving a total of 48 articles eligible for full text reading. 22 studies were excluded for the following reasons: full text was not retrieved (*n* = 5); review (*n* = 8); duplicate articles (*n* = 3); other interventions (*n* = 6). Finally, 25 studies were included in the study. Detailed information on inclusion and exclusion is available in Figure 1.

### 2.2 Study characteristics

We included 25 randomized controlled trials conducted between 2014 and 2022, involving a total of 1,600 women aged between 26 and 28 who had been diagnosed with PCOS according to the Rotterdam criteria. All included studies originated from Iran, indicating a geographically and ethnically homogeneous study population. The intervention kinds included calcium (*n* = 6), magnesium (*n* = 6), selenium (*n* = 7), and chromium (*n* = 6), all of which were taken orally daily as supplements. 200 μg of chromium, 1000 mg of calcium, 250 mg of magnesium and 200 μg of selenium, were the usual amounts. The follow-up period was usually 8–12 weeks long. A placebo was used as the control group in most studies, and factors such as oxidative stress, inflammation, sex hormone levels, insulin resistance, and glucose and lipid metabolism were used to measure the effect of the intervention. Some studies have used a combined supplementation technique (calcium + vitamin D, magnesium + vitamin E, etc.) to look at synergistic benefits. Details of the study’s features are shown in Table 1.

**TABLE 1:** Summary of 25 Supplementation RCTs in Women with PCOS.

Study	Country	PCOS definition	Trial sample size	Control sample size	Trial age	Control age	Trial intervention	Control intervention	Follow-up duration	Main outcomes
Alizadeh et al. (43)	Iran	Rotterdam	52	52	18-40	18-40	Mg 250 mg /d	Placebo	8 weeks	↑Total testosterone, LDL-C, HDL-C and insulin, HOMA-IR.
Asemi et al. (44)	Iran	Rotterdam	26	26	18-40	18-40	Calcium 1000 mg/d + Vit D 50,000 IU/wk	Placebo	8 weeks	↓Insulin, ↓HOMA-IR, ↑QUICKI, ↓TG, ↓VLDL
Ashoush et al. (45)	Iran	Rotterdam	50	50	20-35	20-35	Chromium 200 μg/day	Placebo	6 months	↓FPG, Insulin, TG; ↑QUICKI, HDL
Farsinejad-Marj et al. (46)	Iran	Rotterdam	30	30	26.4±NA	26.4±NA	Mg 250 mg/d	Placebo	8 weeks	↓BMI, ↑DHEA, marginal ↓T, ↑LH
Firouzabadi et al. (47)	Iran	Rotterdam	50	50	26.6±4.4	26.6±4.4	Ca 1000 mg/d + Vit D 50,000 IU/2 wk + Met	Metformin only	8 weeks	↑Menses regularity, ↓BMI, ↓T, ↓HOMA-IR
Foroozanfard et al. (48)	Iran	Rotterdam	45	45	26.2 ± 4.1	26.2 ± 4.1	Ca 1000 mg/d + Vit D 50,000 IU/2 wk + Met	Metformin only	8 weeks	↓BMI, ↓waist, ↑menses regularity
Gholizadeh-Moghaddam et al. (49)	Iran	Rotterdam	32	32	NA	NA	Mg 250 mg /d	Placebo	10 weeks	↓FPG, Insulin, HOMA-IR, TG, TC, LDL; ↑HDL
Heidar et al. (50)	Iran	Rotterdam	18	18	NA	NA	Selenium 200 μg/d	Placebo	8 weeks	↓TC, ↓LDL, ↓Insulin, ↓MDA, ↑TAC
Mohammad Hosseinzadeh et al. (51)	Iran	Rotterdam	30	30	27.1 ± 4.3	26.7 ± 3.8	Chromium 200 μg/day	Placebo	8 weeks	↓CRP, MDA; ↑TAC
Kadoura et al. (52)	Iran	Rotterdam	45	45	27.1 ± 5.3	27.1 ± 5.3	Ca 1000 mg/d + Vit D 50,000 IU/2 wk	Placebo	8 weeks	↓hs-CRP, ↓MDA, ↑TAC
Jamilian et al. (53)	Iran	Rotterdam	30	30	26.3 ± 4.6	26.9 ± 4.8	Chromium 200 μg/day	Placebo	8 weeks	↓Insulin, HOMA-IR, hs-CRP; ↑TAC
Jamilian et al. (54)	Iran	Rotterdam	30	30	27.3 ± 4.5	28.1 ± 4.9	Chromium 200 μg/day	Placebo	8 weeks	↓FPG, Insulin, HOMA-IR, TG, VLDL; ↑QUICKI
Jamilian et al. (55)	Iran	Rotterdam	35	35	18–40	18–40	Selenium 200 μg/day	Placebo	8 weeks	↓Insulin, ↓HOMA-IR, ↓HOMA-B, ↑QUICKI, ↓TG, ↓VLDL
Jamilian et al. (56)	Iran	Rotterdam	30	30	NA	NA	Mg 250 mg + Vit E 400IU	Placebo	12 weeks	↓Insulin, HOMA-IR, hs-CRP, MDA; ↑TAC
Jamilian et al. (57)	Iran	Rotterdam	30	30	26.7 ± 4.5	27.3 ± 4.6	Chromium 200 μg/day	Placebo	8 weeks	↓Testosterone, ↑SHBG
Jamilian et al. (58)	Iran	Rotterdam	30	30	26.4 ± 4.2	27.1 ± 3.9	Chromium picolinate 200 μg twice/day	Placebo	8 weeks	↓FPG, HOMA-IR, TG, VLDL; ↑QUICKI
Zadeh Modarres et al. (59)	Iran	Rotterdam	30	30	NA	NA	Selenium 200 μg/day	Placebo	8 weeks	↓Insulin, ↓HOMA-IR, ↑QUICKI, ↓TG, ↓MDA
Zadeh Modarres et al. (60)	Iran	Rotterdam	30	30	NA	NA	Selenium 200 μg/day	Placebo	8 weeks	↓FAI, ↓hs-CRP, ↓MDA, ↑TAC
Mousavi et al. (61)	Iran	Rotterdam	21	21	NA	NA	Mg 250 mg + Melatonin 3 mg	Placebo	8 weeks	↓Hirsutism, ↓TNF-α, ↑TAC
Rashidi et al. (62)	Iran	Rotterdam	30	30	NA	NA	Selenium 200 μg/day	Placebo	8 weeks	↓ADMA, ↓TC, ↓LDL, ↓Insulin, ↑QUICKI
Razavi et al. (63)	Iran	Rotterdam	32	32	18–40	18–40	Selenium 200 μg/day	Placebo	8 weeks	↑Pregnancy rate, ↓DHEA, ↓hs-CRP, ↓MDA, ↓Alopecia & Acne
Razavi et al. (64)	Iran	Rotterdam	30	30	18-40	18–40	Vit D 200 IU + Vit K2 90 μg + Ca 500 mg, bid	Placebo	8 weeks	↓Free T, ↓DHEAS, ↑TAC, ↓MDA
Shokrpour et al. (65)	Iran	Rotterdam	30	30	NA	NA	Mg (unspecified dose)	Placebo	8 weeks	↓Testosterone, ↑E2, improved hirsutism and sleep
Amiri Siavashani et al. (66)	Iran	Rotterdam	20	20	18–40	18–40	Chromium 200 μg/day	Placebo	8 weeks	hs-CRP,PPAR-γ, GLUT-1, LDLR, IL-1, IL-8, TNF-α, TGF-β, VEGF.
Tehrani et al. (67)	Iran	Rotterdam	45	45	27.2 ± 5.4	27.2 ± 5.4	Ca 1000 mg/d + Vit D 50,000 IU/2 wk	Placebo	8 weeks	↓FINS, ↓HOMA-IR, ↓TC, ↓TG, ↓LDL

The age of the trial group and the control group is expressed as mean ± standard deviation or range. NA, not available. CRP, C-reactive protein; FAI, free androgen index; HDL, high-density lipoprotein; HOMA-IR, homeostasis model assessment of insulin resistance; LDL, low-density lipoprotein; MDA, malondialdehyde; SHBG, sex hormone-binding globulin; TC, total cholesterol; TG, triglycerides; VLDL, very-low-density lipoprotein; DHEA, dehydroepiandrosterone; LH, luteinizing hormone; QUICKI, quantitative insulin sensitivity check index; TAC, total antioxidant capacity; ADMA, asymmetric dimethylarginine; BMI, body mass index; FINS, fasting insulin; FPG, fasting plasma glucose; HOMA-B, homeostasis model assessment of β-cell function; IL, interleukin; TNF, tumor necrosis factor; VEGF, vascular endothelial growth factor.

## 3 Results

### 3.1 Glucose metabolism

#### 3.1.1 Fasting blood glucose (FPG)

As shown in Table 2, pooled analysis from six studies demonstrated that chromium supplementation significantly reduced FPG levels (SMD = −0.79, 95% CI: −1.11 to −0.46, *P* < 0.01). In contrast, selenium supplementation showed no significant effect (SMD = −0.32, 95% CI: −1.02 to 0.39, *P* = 0.38).

**TABLE 2:** The effect of trace elements on blood glucose measures in PCOS patient.

Measures	Calcium	Chromium	Magnesium	Selenium
FPG	/	**−0.79 (−1.11, −0.46)** *P* < 0.01*	/	−0.32 (−1.02, 0.39) *P* = 0.38
Insulin	/	**−0.58 (−0.90, −0.26)** *P* < 0.01*	−0.09 (−0.40, 0.22) *P* = 0.58	**−0.32 (−0.63, −0.01)** *P* = 0.04*
HOMA-IR	/	**−0.68 (−1.00, −0.36)** *P* < 0.01*	−0.10 (−0.41, 0.21) *P* = 0.54	−0.26 (−0.57, 0.05) *P* = 0.11
HOMA-B	/	−0.09 (−0.95, 0.76) *P* = 0.83	0.14 (−0.25, 0.53) *P* = 0.48	/
QUICKI	/	0.74 (−0.05, 1.53) *P* = 0.07	0.49 (−0.48, 1.46) *P* = 0.32	**0.53 (0.15, 0.91)** *P* = 0.01*

*Indicate statistical significance. FPG, Fasting plasma glucose; HOMA-IR, Homeostasis model assessment of Insulin Resistance; HOMA-B, Homeostasis model assessment of β-cell function; QUICKI, Quantitative insulin sensitivity check index.

#### 3.1.2 Fasting insulin

As shown in Table 2, meta-analysis of nine studies revealed significant reductions in fasting insulin following supplementation with chromium (SMD = −0.58, 95% CI: −0.90 to −0.26, *P* < 0.01) and selenium (SMD = −0.32, 95% CI: −0.63 to −0.01, *P* = 0.04). Magnesium supplementation had no significant effect (SMD = −0.09, 95% CI: −0.40 to 0.22, *P* = 0.58).

#### 3.1.3 HOMA-IR

As shown in Table 2, chromium supplementation significantly improved HOMA-IR (SMD = −0.68, 95% CI: −1.00 to −0.36, *P* < 0.01) in nine studies. Neither magnesium (SMD = −0.10, 95% CI: −0.41 to 0.21, *P* = 0.54) nor selenium (SMD = −0.26, 95% CI: −0.57 to 0.05, *P* = 0.11) produced statistically significant changes.

#### 3.1.4 HOMA-B

As shown in Table 2, no significant alterations in HOMA-B were observed with magnesium (SMD = 0.14, 95% CI: −0.25 to 0.53, *P* = 0.48) or chromium (SMD = −0.09, 95% CI: −0.95 to 0.76, *P* = 0.83) supplementation in four studies.

#### 3.1.5 QUICKI

As shown in Table 2, selenium supplementation significantly improved QUICKI values (SMD = 0.53, 95% CI: 0.15 to 0.91, *P* < 0.01). Although not statistically significant, increasing trends were observed for chromium (SMD = 0.74, 95% CI: −0.05 to 1.53, *P* = 0.07) and magnesium (SMD = 0.49, 95% CI: −0.48 to 1.46, *P* = 0.32).

### 3.2 Lipid metabolism

#### 3.2.1 Triglycerides (TG)

The summarized results are presented in Table 3, chromium supplementation significantly reduced TG levels (SMD = −0.59, 95% CI: −0.91 to −0.27, *P* < 0.01). No significant effects were found for magnesium (SMD = 0.04, 95% CI: −0.84 to 0.92, *P* = 0.93) or selenium (SMD = −0.11, 95% CI: −0.83 to 0.61, *P* = 0.76).

**TABLE 3:** The effect of trace elements on blood lipid measures in PCOS patient.

Measures	Calcium	Chromium	Magnesium	Selenium
Triglycerides	/	**−0.59 (−0.91, −0.27)** *P* < 0.01*	0.04 (−0.84, 0.92) *P* = 0.93	−0.11 (−0.83, 0.61) *P* = 0.76
Total cholesterol	/	−0.32 (−0.36, −0.00) *P* = 0.05	−0.11 (−0.42, 0.20) *P* = 0.50	−0.24 (−0.54, 0.06) *P* = 0.117
VLDL	/	**−0.59 (−0.91, −0.27)** *P* < 0.01*	/	**−0.39 (−0.76, −0.01)** *P* = 0.04*
HDL	/	0.02 (−0.37, 0.40) *P* = 0.93	−0.07 (−0.38, 0.24) *P* = 0.65	−0.20 (−0.50, 0.10) *P* = 0.19
LDL	/	−0.13 (−0.45, 0.18) *P* = 0.40	−0.03 (−0.34, 0.28) *P* = 0.83	−0.14 (−0.44, 0.16) *P* = 0.35

*Indicate statistical significance. VLDL, very-low-density lipoprotein; LDL, low-density lipoprotein; HDL, high-density lipoprotein.

#### 3.2.2 Total cholesterol (TC)

The summarized results are presented in Table 3, none of the supplements produced a significant reduction in TC levels (Chromium: SMD = −0.32, 95% CI: −0.63 to 0.00, *P* = 0.05; Magnesium: SMD = −0.11, 95% CI: −0.42 to 0.20, *P* = 0.50; Selenium: SMD = −0.24, 95% CI: −0.54 to 0.06, *P* = 0.11).

#### 3.2.3 VLDL

The summarized results are presented in Table 3, both chromium (SMD = −0.59, 95% CI: −0.91 to −0.27, *P* < 0.01) and selenium (SMD = −0.39, 95% CI: −0.76 to −0.01, *P* = 0.05) supplementation significantly reduced VLDL levels.

#### 3.2.4 HDL & LDL

The summarized results are presented in Table 3, no supplementation significantly altered HDL or LDL levels (all *P* > 0.05).

### 3.3 Sex hormones and androgens

The effects of trace element supplementation on sex hormone - related measures are detailed in Table 4. Supplementation with calcium, magnesium, or selenium did not significantly alter serum levels of LH, FSH, DHEA, SHBG, testosterone (total or serum), or the Free Androgen Index (FAI) (all *P* > 0.05).

**TABLE 4:** The effect of trace elements on blood sex hormone-related measures in PCOS patient.

Measures	Calcium	Chromium	Magnesium	Selenium
LH	−0.55 (−1.15, 0.05) *P* = 0.07	/	/	/
FSH	−0.28 (−0.70, 0.14) *P* = 0.19	/	/	/
DHEA	/	/	−12.19 (−37.57, 13.19) *P* = 0.35	/
SHBG	/	/	0.20 (−0.11, 0.51) *P* = 0.21	0.20 (−0.16, 0.56) *P* = 0.28
Testosterone	/	/	0.23 (−0.16, 0.63) *P* = 0.25	/
Total testosterone	/	/	/	0.13 (−0.23, 0.49) *P* = 0.47
FAI	/	/	0.14 (−0.17, 0.45) *P* = 0.38	/

*Indicate statistical significance. FSH, follicle stimulating hormone; LH, luteinizing hormone; DHEA, dehydroepiandrosterone; SHBG, sex hormone binding globulin; FAI, free androgen index.

### 3.4 Oxidative stress and inflammation

#### 3.4.1 MDA

As indicated in Table 5, calcium (SMD = −0.76, 95% CI: −1.15 to −0.36, *P* = 0.0002) and chromium (SMD = −1.69, 95% CI: −3.10 to −0.28, *P* = 0.02) supplementation significantly reduced MDA levels, whereas magnesium and selenium did not.

**TABLE 5:** The effect of trace elements on oxidative stress and inflammation related measures in PCOS patient.

Measures	Calcium	Chromium	Magnesium	Selenium
MDA	**−0.76 (−1.15, −0.36)** *P* < 0.01*	**−1.69 (−3.10, −0.28)** *P* = 0.02*	0.25 (−0.14, 0.64) *P* = 0.21	−0.91 (−1.94, 0.12) *P* = 0.08
NO	**−0.45 (−0.84,−0.06)** *P* = 0.02*	−0.06 (−1.23, 1.11) *P* = 0.92	/	/
GSH	0.51 (−1.29, 2.30) *P* = 0.58	0.34 (−0.05, 0.74) *P* = 0.09	/	0.01 (−0.38, 0.39) *P* = 0.97
TAC	0.52 (−0.75, 1.80) *P* = 0.42	**1.47 (1.02, 1.92)** *P* < 0.01*	/	**0.55 (0.16, 0.95)** *P* < 0.01*
Hs-CRP	0.12 (−0.76, 1.01) *P* = 0.79	**−0.65 (−1.05, −0.24)** *P* < 0.01*	/	/

*Indicate statistical significance. MDA, malondialdehyde; NO, nitric oxide; GSH, glutathione; Hs-CRP, high-sensitivity c-reactive protein; TAC, total antioxidant capacity.

#### 3.4.2 NO

As indicated in Table 5, calcium significantly increased NO levels (SMD = −0.45, 95% CI: −0.84 to −0.06, *P* = 0.02); chromium had no effect.

#### 3.4.3 GSH

As indicated in Table 5, no supplementation significantly increased GSH levels.

#### 3.4.4 TAC

As indicated in Table 5, selenium (SMD = 0.55, 95% CI: 0.16 to 0.95, *P* < 0.01) and chromium (SMD = 1.47, 95% CI: 1.02 to 1.92, *P* < 0.01) supplementation significantly increased TAC.

#### 3.4.5 Hs-CRP

As indicated in Table 5, chromium significantly reduced hs-CRP levels (SMD = −0.65, 95% CI: −1.05 to −0.24, *P* < 0.01); calcium did not.

### 3.5 Anthropometric measures

As shown in Table 6, no trace element supplementation significantly affected BMI, body weight, waist circumference, or hip circumference (all *P* > 0.05).

**TABLE 6:** The effect of trace elements on weight-related measures in PCOS patient.

Measures	Calcium	Chromium	Magnesium	Selenium
BMI	−0.18 (−0.44, 0.09) *P* = 0.19	−0.09 (−0.32, 0.15) *P* = 0.46	−0.04 (−0.35, 0.27) *P* = 0.82	−0.18 (−0.47, 0.10) *P* = 0.21
Body weight	−0.28 (−0.71, 0.15) *P* = 0.20	0.05 (−0.27, 0.36) *P* = 0.77	−0.07 (−0.33, 0.20) *P* = 0.63	−0.08 (−0.40, 0.24) *P* = 0.61
Waist circumference	−0.28 (−0.70, 0.15) *P* = 0.21	/	−0.13 (−0.52, 0.26) *P* = 0.52	/
Hip circumference	−0.30 (−0.73, 0.13) *P* = 0.17	/	/	/

*Indicate statistical significance. BMI, body mass index.

## 4 Discussion

### 4.1 Main findings

This meta-analysis included 25 RCTs with 1,600 patients with PCOS and systematically assessed the effects of four trace elements: calcium, chromium, selenium and magnesium on oxidative stress and inflammatory responses, glucose and lipid metabolism, sex hormone levels, and weight-related parameters. Chromium may be a better supplement for PCOS metabolic therapy, as the results indicate that it has the most beneficial effects on oxidative stress, dysregulated glucose and lipid metabolism, and other metabolic problems.

Calcium and selenium have complementary benefits by enhancing antioxidant defenses and somewhat raising insulin sensitivity, respectively. In this study, magnesium did not provide any appreciable therapeutic advantages. However, no single trace element had a substantial effect on either weight management or sex hormone regulation, suggesting that future research should look at strategies based on the coordinated intervention of many dietary components.

This study systematically validated and expanded on previous studies on the intervention effects of trace elements on oxidative stress and metabolic parameters associated with PCOS. By synthesizing the corpus of prior information, it also made clear the distinct functions and processes of certain trace elements. First, chromium has been shown to have beneficial effects on glucose metabolism, as evidenced by improvements in key indicators such as FPG and the HOMA-IR (14). The beneficial effects of chromium on lipid metabolism were quantified in this study. These effects included modulatory effects on oxidative stress markers such as MDA, TAC, and Hs-CRP, as well as decreases in TG and VLDL (20). This study provides a more comprehensive line of data supporting the multi-targeted intervention of chromium in PCOS. Furthermore, despite contradictory findings from past research on the mineral’s role in insulin regulation, recent studies suggest that selenium may enhance the QUICKI and TAC (21). It also has potential as a therapy to increase insulin sensitivity (22). Although selenium supplementation was associated with a substantial rise in the quantitative insulin sensitivity check index and TAC, it did not significantly lower FPG or the homeostatic model assessment of insulin resistance, according to this study’s detailed analysis (23). These results suggest that the main way that selenium may influence metabolism is via indirectly increasing the body’s antioxidant capacity, which improves intracellular This work fully investigates the effects of magnesium on metabolic regulation, and the body of evidence from previous research indicates that magnesium has a positive regulatory influence on insulin sensitivity (24).

However, even after integrating a larger number of RCTs, this study did not identify any significant improvements in sex hormone levels or glucose and lipid metabolism associated with magnesium intake. These findings raise doubts about the therapeutic efficacy of magnesium as a stand-alone supplementary therapy. Furthermore, some studies have demonstrated that magnesium supplementation by alone does not improve PCOS-related markers. This suggests that magnesium may need to cooperate with other nutrients in order to have a substantial metabolic or hormonal impact (25).

Polycystic ovarian syndrome is a prevalent endocrine disorder in women that is frequently associated with significant elements of the metabolic syndrome, including obesity, dyslipidemia, and insulin resistance (26). Patients with PCOS often have disruptions in the insulin signaling pathway, which can manifest clinically as hyperinsulinemia and insulin resistance (27). Chromium enhances the efficiency of glucose absorption and utilization via increasing insulin receptor activation and the PI3K/Akt signaling pathway (28). Selenium raises insulin sensitivity due to its antioxidant properties and capacity to support enhanced signal transduction (29). Since magnesium is a cofactor required for insulin signaling, it has a positive link with insulin sensitivity (30). In this case, altering calcium ion channels is essential because disruption of calcium homeostasis may exacerbate insulin resistance (31). Enhancing lipid metabolism, trace element supplementation alters the production and clearance of cholesterol and triglycerides by directly affecting the expression and activity of fatty acid metabolic enzymes *in vivo*. Chromium increases the activation of AMPK and PPAR-α signaling pathways to reduce hepatic triglyceride accumulation and boost fatty acid β-oxidation (32). Magnesium aids in controlling the metabolic balance of fatty acids as a required cofactor for a number of enzymes involved in lipid metabolism, including lipase and acetyl-CoA carboxylase (33). As a crucial part of glutathione peroxidase, selenium facilitates the efficient removal of lipid peroxidation products, such as lipid hydroperoxides, as several review articles have highlighted (34, 35). Calcium effectively inhibits the oxidation of LDL. Furthermore, in the gastrointestinal tract, calcium may form insoluble calcium soaps with dietary fatty acids, which reduce intestinal absorption of triglycerides and cholesterol (36). One of the most important pathophysiological processes behind PCOS is oxidative stress (37). As a required cofactor of glutathione peroxidase, selenium helps the body produce a significant amount of TAC (23). By stabilizing mitochondrial activity and regulating intracellular calcium signaling, magnesium demonstrates antioxidant potential (38), however its efficacy depends on concurrent supplementation with other nutrients (39).

This meta-analysis and systematic review has a number of important advantages. First, as far as we are aware, this study provides the most thorough synthesis to date of the effects of four common trace elements on PCOS patients: calcium, chromium, selenium and magnesium. Supported by a multidimensional coverage and a well-structured assessment methodology, the study covers a wide variety of fundamental outcome indicators, such as oxidative stress markers, body weight management, sex hormone profiles, and glucose and lipid metabolism. Second, the PRISMA declaration was strictly followed in the conduct of this study. Since all of the included studies are RCTs, they exhibit a high degree of methodological rigor and offer solid proof to back up clinical judgment. This study does, however, have a number of drawbacks. First, there is significant variation in the included studies’ intervention dose, duration, and administration methods, which might jeopardize the findings’ generalizability and robustness. Second, there is a small sample size overall, which means that certain results have a low statistical power and emphasizes the necessity of validation through bigger, more powerful studies. Thirdly, the studies we included were all from Iran, which to some extent affects the generalizability of our results. Fourth, the observed effects of the intervention may have been influenced by confounding factors because a number of the included trials lacked long-term follow-up evaluations and did not account for lifestyle characteristics. This restriction limits how broadly the results may be applied. In conclusion, this study offers important data about the metabolic regulating potential of trace elements in PCOS patients, notwithstanding these limitations. To support these conclusions, more excellent research with bigger sample numbers and standardized intervention procedures is required.

Polycystic ovarian syndrome is associated with an increased risk profile for cardiovascular diseases. PCOS is a risk-enhancing illness that necessitates more regular cardiovascular risk assessment and monitoring, according to the clinical practice guidelines of the American Heart Association and the American College of Cardiology. Long-term medical interventions are crucial to the therapeutic management of PCOS because they lower the risk of cardiovascular diseases. Recent worldwide evidence-based guidelines for PCOS care place a strong focus on routine monitoring of key metabolic indicators, including blood glucose and lipid profiles (40). Emerging research indicates that micronutrients and other nutritional supplements provide experimental therapeutic options for PCOS, with early results showing potential advantages in managing the illness (41). Because trace minerals have antioxidant properties that might lower oxidative stress, they may be employed as therapeutic agents to treat PCOS (42). Second, the effects of trace elements on metabolic profiles in different PCOS phenotypes are not well understood at this time. Therefore, properly designed RCTs with adequately powered sample sizes are needed to completely evaluate the impact of trace elements on key PCOS features.

## Data Availability

The original contributions presented in this study are included in this article/Supplementary material, further inquiries can be directed to the corresponding author.
