# Supplementary material for: The effects of trace element supplementation on glycolipid metabolism in PCOS: a systematic review and meta-analysis
Source: Front Nutr. 2025 Oct 9;12:1683556. doi: 10.3389/fnut.2025.1683556 (PMC12545135; doi:10.3389/fnut.2025.1683556)
Supplement: Supplementary file 1 [file Table_1.docx]

**Detailed search strategy**

PUBMED (Searched on: May 1,2025)

| Search number | Query | Results | Time |
| --- | --- | --- | --- |
| #1 | ((((Trace element[Title/Abstract]) OR (Calcium[Title/Abstract])) OR (chromium[Title/Abstract])) OR (magnesium[Title/Abstract])) OR (selenium[Title/Abstract]) | 726,102 | 18:30:03 |
| #2 | (((((((polycystic ovarian syndrome[MeSH Terms]) OR (polycystic ovarian syndrome[Title/Abstract])) OR (Polycystic Ovary Syndrome[Title/Abstract])) OR (Ovary Syndrome[Title/Abstract])) OR (Polycystic Syndrome[Title/Abstract])) OR (Polycystic Ovary[Title/Abstract])) OR (Polycystic ovary disease[Title/Abstract])) OR (PCOS[Title/Abstract]) | 28,133 | 18:32:22 |
| #3 | ((((Randomized controlled trial[Publication Type]) OR (Randomized controlled trial[Title/Abstract])) OR (Randomized[Title/Abstract])) OR (Placebo[Title/Abstract])) OR (Random[Title/Abstract]) | 1,495,791 | 18:33:01 |
| #4 | #1 AND #2 AND #3 | 97 | 18:34:55 |

EMBASE (Searched on: May 1,2025)

| Search number | Query | Results | Time |
| --- | --- | --- | --- |
| #1 | 'trace element':ab,ti OR 'calcium':ab,ti OR 'chromium':ab,ti OR 'magnesium':ab,ti OR 'selenium':ab,ti | 876,928 | 19:12:11 |
| #2 | 'ovary polycystic disease'/exp OR 'polycystic ovarian syndrome':ti,ab,kw OR 'polycystic ovary syndrome':ti,ab,kw OR 'ovary syndrome':ti,ab,kw OR 'polycystic syndrome':ti,ab,kw OR 'polycystic ovary':ti,ab,kw OR 'polycystic ovary disease':ti,ab,kw OR pcos:ti,ab,kw | 50,183 | 19:17:23 |
| #3 | 'randomized controlled trial'/exp OR 'randomized controlled trial':ab,ti OR 'rct':ab,ti OR 'random':ab,ti OR 'placebo':ab,ti | 1,839,155 | 19:18:13 |
| #4 | #1 AND #2 AND #3 | 145 | 19:22:09 |

WOS (Searched on: May 1,2025)

| Search number | Query | Results | Time |
| --- | --- | --- | --- |
| #1 | (((((TS=(Trace element)) OR TS=(Calcium)) OR TS=(chromium)) OR TS=(magnesium)) OR TS=(selenium)) | [2,666,932](http://webofscience-clarivate-cn-s.vpn1.hactcm.edu.cn/wos/alldb/summary/ef1def11-9577-4490-9bc7-a71e6f0720c8-016c1362af/relevance/1) | 20:32:07 |
| #2 | ((((((TS=(polycystic ovarian syndrome)) OR TS=(Polycystic Ovary Syndrome)) OR TS=(Ovary Syndrome)) OR TS=(Polycystic Syndrome)) OR TS=(Polycystic Ovary)) OR TS=(Polycystic ovary disease)) OR TS=(PCOS) | [58,657](http://webofscience-clarivate-cn-s.vpn1.hactcm.edu.cn/wos/alldb/summary/fdf30b2b-233f-4e0b-8376-dfba999a561d-016c136b0b/relevance/1) | 20:38:42 |
| #3 | ((((TS=(randomized controlled trial)) OR TS=(RCT)) OR TS=(random)) OR TS=(randomized)) OR TS=(placebo) | [2,985,949](http://webofscience-clarivate-cn-s.vpn1.hactcm.edu.cn/wos/alldb/summary/0812d8fa-66e7-4ef5-bdf6-b1fa60f5eafd-016c136d66/relevance/1) | 20:50:37 |
| #4 | #1 AND #2 AND #3 | 232 | 20:58:22 |

Cochrane (Searched on: May 1,2025)

| Search number | Query | Results | Time |
| --- | --- | --- | --- |
| #1 | (trace element):ti,ab,kw OR (calcium):ti,ab,kw OR (chromium):ti,ab,kw OR (magnesium):ti,ab,kw OR (selenium):ti,ab,kw | 52,685 | 21:33:22 |
| #2 | (polycystic ovarian syndrome):ti,ab,kw OR (Polycystic Ovary Syndrome):ti,ab,kw OR (Ovary Syndrome):ti,ab,kw OR (Polycystic Syndrome):ti,ab,kw OR (Polycystic Ovary):ti,ab,kw OR (Polycystic ovary disease):ti,ab,kw OR (PCOS):ti,ab,kw | 6,614 | 21:38:42 |
| #3 | (randomized controlled trial):ti,ab,kw OR (randomized controlled trial):ti,ab,kw OR (rct):ti,ab,kw OR (random):ti,ab,kw OR (placebo):ti,ab,kw | 1,077,203 | 21:48:24 |
| #4 | #1 AND #2 AND #3 | 182 | 21:50:20 |
