# Supplementary material for: The effects of trace element supplementation on glycolipid metabolism in PCOS: a systematic review and meta-analysis
Source: Front Nutr. 2025 Oct 9;12:1683556. doi: 10.3389/fnut.2025.1683556 (PMC12545135; doi:10.3389/fnut.2025.1683556)
Supplement: Supplementary file 2 [file Table_2.docx]

Bias graph for randomized controlled trials.

| **Study** | **Random sequence generation** | **Allocation concealment** | **Blinding of participants and personnel** | **Blinding of outcome date** | **Incomplete outcome assessment** | **Selective reporting** | **Other bias** |
| --- | --- | --- | --- | --- | --- | --- | --- |
| Alizadeh2021**(1)** | Low | Unclear | Unclear | Unclear | Low | Low | Low |
| Asemi 2014**(2)** | Low | Unclear | Low | Unclear | Low | Low | Low |
| Ashoush2016(3) | Low | Unclear | Low | Unclear | Low | Low | Low |
| Farsinejad-Marj 2020(4) | Low | Unclear | Low | Low | Low | Low | Low |
| Firouzabadi2012(5) | Low | High | High | Unclear | Low | Low | Low |
| Foroozanfard 2015(6) | Low | Unclear | Low | Unclear | Low | Low | Low |
| Moghaddam2022(7) | Low | Low | Low | Unclear | Low | Low | Low |
| Heidar2019(8) | Low | Low | Low | Unclear | Low | Low | Low |
| Hosseinzadeh 2016(9) | Low | Low | Low | Unclear | Low | Low | Low |
| Kadoura 2019(10) | Low | Low | Low | Unclear | Low | Low | Low |
| Jamilian 2015(11) | Low | Low | Low | Unclear | Unclear | Low | Low |
| Jamilian 2015b(12) | Low | Low | Unclear | Unclear | Unclear | Low | Low |
| Jamilian 2015c(13) | Low | Low | Unclear | Unclear | Low | Low | Low |
| Jamilian 2018(14) | Low | Low | Low | Unclear | Low | Low | Low |
| Jamilian2018b(15) | Low | Low | Low | Unclear | Unclear | Low | Low |
| Jamilian 2019(16) | Low | Low | Low | Low | Low | Low | Low |
| Modarres 2017(17) | Low | Low | Low | Low | Low | Low | Low |
| Modarres2022(18) | Low | Low | Low | Low | Unclear | Low | Low |
| Mousavi 2021(19) | Low | Low | Unclear | Unclear | Unclear | Low | Low |
| Rashidi 2019(20) | Low | Low | Low | Low | Unclear | Low | Low |
| Razavi 2015(21) | Low | Low | Low | Low | Low | Low | Low |
| Razavi 2016(22) | Low | Low | Low | Low | Unclear | Low | Low |
| Shokrpour 2019(23) | Low | Unclear | Unclear | Unclean | Low | Low | Low |
| Siavashani 2018(24) | Low | Unclear | Low | Unclear | Low | Low | Low |
| Tehrani 2014(25) | Low | Unclear | Low | Unclear | Low | Low | Low |

Low: Low of bias; high: high of bias; unclear: unclear of bias.

**References：**

1. Alizadeh M, Karandish M, Asghari Jafarabadi M, et al. Metabolic and Hormonal Effects of Melatonin and/or Magnesium Supplementation in Women with Polycystic Ovary Syndrome: A Randomized, Double-Blind, Placebo-Controlled Trial. *Nutrition & Metabolism*, 2021, 18(1): 57. doi:10.1186/s12986-021-00586-9.

2. Asemi Z, Foroozanfard F, Hashemi T, et al. Calcium plus Vitamin D Supplementation Affects Glucose Metabolism and Lipid Concentrations in Overweight and Obese Vitamin D Deficient Women with Polycystic Ovary Syndrome. *Clinical Nutrition (edinburgh, Scotland)*, 2015, 34(4): 586–592. doi:10.1016/j.clnu.2014.09.015.

3. Ashoush S, Abou-Gamrah A, Bayoumy H, et al. Chromium picolinate reduces insulin resistance in polycystic ovary syndrome: randomized controlled trial. *Journal of Obstetrics and Gynaecology Research*, 2016, 42(3): 279–285. doi:10.1111/jog.12907.

4. Farsinejad-Marj M, Azadbakht L, Mardanian F, et al. Clinical and Metabolic Responses to Magnesium Supplementation in Women with Polycystic Ovary Syndrome. *Biological Trace Element Research*, 2020, 196(2): 349–358. doi:10.1007/s12011-019-01923-z.

5. Firouzabadi R dehghani, Aflatoonian A, Modarresi S, et al. Therapeutic Effects of Calcium & Vitamin D Supplementation in Women with PCOS. *Complementary Therapies in Clinical Practice*, 2012, 18(2): 85–88. doi:10.1016/j.ctcp.2012.01.005.

6. Foroozanfard F, Jamilian M, Bahmani F, et al. Calcium plus Vitamin D Supplementation Influences Biomarkers of Inflammation and Oxidative Stress in Overweight and Vitamin D-Deficient Women with Polycystic Ovary Syndrome: A Randomized Double-Blind Placebo-Controlled Clinical Trial. *Clinical Endocrinology*, 2015, 83(6): 888–894. doi:10.1111/cen.12840.

7. Gholizadeh-Moghaddam M, Ghasemi-Tehrani H, Askari G, et al. Effect of Magnesium Supplementation in Improving Hyperandrogenism, Hirsutism, and Sleep Quality in Women with Polycystic Ovary Syndrome: A Randomized, Placebo-Controlled Clinical Trial. *Health Science Reports*, 2023, 6(1): e1013. doi:10.1002/hsr2.1013.

8. Heidar Z, Hamzepour N, Zadeh Modarres S, et al. The Effects of Selenium Supplementation on Clinical Symptoms and Gene Expression Related to Inflammation and Vascular Endothelial Growth Factor in Infertile Women Candidate for In Vitro Fertilization. *Biological Trace Element Research*, 2020, 193(2): 319–325. doi:10.1007/s12011-019-01715-5.

9. Mohammad Hosseinzadeh F, Hosseinzadeh-Attar M J, Yekaninejad M S, et al. Effects of Selenium Supplementation on Glucose Homeostasis and Free Androgen Index in Women with Polycystic Ovary Syndrome: A Randomized, Double Blinded, Placebo Controlled Clinical Trial. *Journal of Trace Elements in Medicine and Biology: Organ of the Society for Minerals and Trace Elements (GMS)*, 2016, 34: 56–61. doi:10.1016/j.jtemb.2016.01.002.

10. Kadoura S, Alhalabi M, Nattouf A H. Effect of Calcium and Vitamin D Supplements as an Adjuvant Therapy to Metformin on Menstrual Cycle Abnormalities, Hormonal Profile, and IGF-1 System in Polycystic Ovary Syndrome Patients: A Randomized, Placebo-Controlled Clinical Trial. *Advances in Pharmacological Sciences*, 2019, 2019: 9680390. doi:10.1155/2019/9680390.

11. Jamilian M, Bahmani F, Siavashani M A, et al. The Effects of Chromium Supplementation on Endocrine Profiles, Biomarkers of Inflammation, and Oxidative Stress in Women with Polycystic Ovary Syndrome: A Randomized, Double-Blind, Placebo-Controlled Trial. *Biological Trace Element Research*, 2016, 172(1): 72–78. doi:10.1007/s12011-015-0570-6.

12. Jamilian M, Asemi Z. Chromium Supplementation and the Effects on Metabolic Status in Women with Polycystic Ovary Syndrome: A Randomized, Double-Blind, Placebo-Controlled Trial. *Annals of Nutrition & Metabolism*, 2015, 67(1): 42–48. doi:10.1159/000438465.

13. Jamilian M, Razavi M, Fakhrie Kashan Z, et al. Metabolic Response to Selenium Supplementation in Women with Polycystic Ovary Syndrome: A Randomized, Double-Blind, Placebo-Controlled Trial. *Clinical Endocrinology*, 2015, 82(6): 885–891. doi:10.1111/cen.12699.

14. Jamilian M, Sabzevar N K, Asemi Z. The Effect of Magnesium and Vitamin E Co-Supplementation on Glycemic Control and Markers of Cardio-Metabolic Risk in Women with Polycystic Ovary Syndrome: A Randomized, Double-Blind, Placebo-Controlled Trial. *Hormone and Metabolic Research = Hormon- Und Stoffwechselforschung = Hormones Et Metabolisme*, 2019, 51(2): 100–105. doi:10.1055/a-0749-6431.

15. Jamilian M, Zadeh Modarres S, Amiri Siavashani M, et al. The Influences of Chromium Supplementation on Glycemic Control, Markers of Cardio-Metabolic Risk, and Oxidative Stress in Infertile Polycystic Ovary Syndrome Women Candidate for In Vitro Fertilization: A Randomized, Double-Blind, Placebo-Controlled Trial. *Biological Trace Element Research*, 2018, 185(1): 48–55. doi:10.1007/s12011-017-1236-3.

16. Jamilian M, Foroozanfard F, Kavossian E, et al. Effects of Chromium and Carnitine Co-Supplementation on Body Weight and Metabolic Profiles in Overweight and Obese Women with Polycystic Ovary Syndrome: A Randomized, Double-Blind, Placebo-Controlled Trial. *Biological Trace Element Research*, 2020, 193(2): 334–341. doi:10.1007/s12011-019-01720-8.

17. Zadeh Modarres S, Heidar Z, Foroozanfard F, et al. The Effects of Selenium Supplementation on Gene Expression Related to Insulin and Lipid in Infertile Polycystic Ovary Syndrome Women Candidate for In Vitro Fertilization: A Randomized, Double-Blind, Placebo-Controlled Trial. *Biological Trace Element Research*, 2018, 183(2): 218–225. doi:10.1007/s12011-017-1148-2.

18. Zadeh Modarres S, Asemi Z, Heidar Z. The Effects of Selenium Supplementation on Glycemic Control, Serum Lipoproteins and Biomarkers of Oxidative Stress in Infertile Women Diagnosed with Polycystic Ovary Syndrome Undergoing in Vitro Fertilization: A Randomized, Double-Blind, Placebo-Controlled Trial. *Clinical Nutrition ESPEN*, 2022, 51: 92–96. doi:10.1016/j.clnesp.2022.07.017.

19. Mousavi R, Alizadeh M, Asghari Jafarabadi M, et al. Effects of Melatonin and/or Magnesium Supplementation on Biomarkers of Inflammation and Oxidative Stress in Women with Polycystic Ovary Syndrome: A Randomized, Double-Blind, Placebo-Controlled Trial. *Biological Trace Element Research*, 2022, 200(3): 1010–1019. doi:10.1007/s12011-021-02725-y.

20. Rashidi B H, Mohammad Hosseinzadeh F, Alipoor E, et al. Effects of Selenium Supplementation on Asymmetric Dimethylarginine and Cardiometabolic Risk Factors in Patients with Polycystic Ovary Syndrome. *Biological Trace Element Research*, 2020, 196(2): 430–437. doi:10.1007/s12011-019-01954-6.

21. Razavi M, Jamilian M, Kashan Z F, et al. Selenium Supplementation and the Effects on Reproductive Outcomes, Biomarkers of Inflammation, and Oxidative Stress in Women with Polycystic Ovary Syndrome. *Hormone and Metabolic Research = Hormon- Und Stoffwechselforschung = Hormones Et Metabolisme*, 2016, 48(3): 185–190. doi:10.1055/s-0035-1559604.

22. Razavi M, Jamilian M, Karamali M, et al. The Effects of Vitamin D-K-Calcium Co-Supplementation on Endocrine, Inflammation, and Oxidative Stress Biomarkers in Vitamin D-Deficient Women with Polycystic Ovary Syndrome: A Randomized, Double-Blind, Placebo-Controlled Trial. *Hormone and Metabolic Research = Hormon- Und Stoffwechselforschung = Hormones Et Metabolisme*, 2016, 48(7): 446–451. doi:10.1055/s-0042-104060.

23. Shokrpour M, Asemi Z. The Effects of Magnesium and Vitamin E Co-Supplementation on Hormonal Status and Biomarkers of Inflammation and Oxidative Stress in Women with Polycystic Ovary Syndrome. *Biological Trace Element Research*, 2019, 191(1): 54–60. doi:10.1007/s12011-018-1602-9.

24. Amiri Siavashani M, Zadeh Modarres S, Mirhosseini N, et al. The Effects of Chromium Supplementation on Gene Expression of Insulin, Lipid, and Inflammatory Markers in Infertile Women with Polycystic Ovary Syndrome Candidate for in Vitro Fertilization: A Randomized, Double-Blinded, Placebo-Controlled Trial. *Frontiers in endocrinology*, 2018, 9: 726. doi:10.3389/fendo.2018.00726.

25. Tehrani H G, Mostajeran F, Shahsavari S. The Effect of Calcium and Vitamin D Supplementation on Menstrual Cycle, Body Mass Index and Hyperandrogenism State of Women with Poly Cystic Ovarian Syndrome. *Journal of Research in Medical Sciences: the Official Journal of Isfahan University of Medical Sciences*, 2014, 19(9): 875–880.
